# Meta-analysis of host response networks identifies a *common core* in tuberculosis

**DOI:** 10.1038/s41540-017-0005-4

**Published:** 2017-02-10

**Authors:** Awanti Sambarey, Abhinandan Devaprasad, Priyanka Baloni, Madhulika Mishra, Abhilash Mohan, Priyanka Tyagi, Amit Singh, JS Akshata, Razia Sultana, Shashidhar Buggi, Nagasuma Chandra

**Affiliations:** 1 Molecular Biophysics Unit, Indian Institute of Science, Bangalore, 560012 Karnataka India; 2 Department of Biochemistry, Indian Institute of Science, Bangalore, 560012 Karnataka India; 3 Centre for Infectious Disease Research, Indian Institute of Science, Bangalore, 560012 Karnataka India; 4 SDS TRC and Rajiv Gandhi Institute of Chest Diseases, Bangalore, 560029 Karnataka India

## Abstract

Tuberculosis remains a major global health challenge worldwide, causing more than a million deaths annually. To determine newer methods for detecting and combating the disease, it is necessary to characterise global host responses to infection. Several high throughput *omics* studies have provided a rich resource including a list of several genes differentially regulated in tuberculosis. An integrated analysis of these studies is necessary to identify a unified response to the infection. Such data integration is met with several challenges owing to platform dependency, patient heterogeneity, and variability in the extent of infection, resulting in little overlap among different datasets. Network-based approaches offer newer alternatives to integrate and compare diverse data. In this study, we describe a meta-analysis of host’s whole blood transcriptomic profiles that were integrated into a genome-scale protein–protein interaction network to generate response networks in active tuberculosis, and monitor their behaviour over treatment. We report the emergence of a highly active *common core* in disease, showing partial reversals upon treatment. The core comprises 380 genes in which *STAT1*, phospholipid scramblase 1 (*PLSCR1*), *C1QB*, *OAS1*, *GBP2* and *PSMB9* are prominent hubs. This network captures the interplay between several biological processes including pro-inflammatory responses, apoptosis, complement signalling, cytoskeletal rearrangement, and enhanced cytokine and chemokine signalling. The *common core* is specific to tuberculosis, and was validated on an independent dataset from an Indian cohort. A network-based approach thus enables the identification of common regulators that characterise the molecular response to infection, providing a platform-independent foundation to leverage maximum insights from available clinical data.

## Tuberculosis: an underlying common-core host response network

Patients suffering from tuberculosis (TB) show a high extent of variations in their gene expression patterns. Such heterogeneity poses major road blocks to our understanding of how hosts respond to the disease. A number of studies have profiled transcriptomes of human blood samples from TB patients, but a meta-analysis indicates that very few changes are consistently seen. The problem, to a large extent, lies with the way large data is analysed. We have used a genome-wide network approach to characterise the host response and have identified a *common-core* in the TB response networks of different patients, indicating the presence of unified host response mechanisms. This core network provides a comprehensive view into the most significant regulators of the infection-mediated biological processes across patients from different populations, and it shows partial reversals upon treatment.

## Introduction

Tuberculosis (TB) is the leading cause of death due to an infectious agent, and continues to pose a global health challenge worldwide. About one-third of the world’s population is estimated to be exposed to *Mycobacterium tuberculosis (Mtb),* with a majority of individuals carrying the bacilli in a latent form. In 2014 alone, approximately 9.6 million people were estimated to have acquired TB, resulting in 1.5 million deaths.^1^ Over time, *Mtb* has evolved several immune evasion strategies that enable it to reside successfully in the host, resulting either in the manifestation of active disease or latent infection.^2, 3^ It has been well recognised that the outcome of infection is a result of complex dynamics between the host and the pathogen, triggering a series of signalling cascades and cross-talk among various molecular components.^4^ The importance of several innate immune responses in clearing tuberculosis infection has been well established, although their relative contribution in a quantitative sense towards mounting adequate adaptive immune responses still remain poorly understood. Decades of genetic and biochemical experimental studies on TB have identified many important factors that contribute to a disease phenotype,^5, 6^ with studies reporting genetic polymorphisms in pattern recognition receptors such as, the mannose receptor^7^ and Toll-like receptors,^8^ several cytokines, chemokines,^9, 10^ *LTA4H*,^11^ *VDR*,^12^ as well as the identification of genetic loci associated with increased risk to TB.^13^ Independent analyses have identified processes of both innate and adaptive immune responses as well as metabolic processes to be significantly implicated in the disease.^6, 14, 15^ However, biological systems are inherently interconnected and interdependent, and rarely will an alteration in a single gene or gene product directly result in disease. Disease states are in fact representations of perturbations in the underlying complex interdependent molecular networks. It is thus important to adopt a systems approach to address this fundamental complexity. These approaches result in novel emergent properties that are difficult to comprehend without considering the whole system.

Recent years have seen a surge in the availability of *omics* data for the host in TB at different levels, furthering our understanding of factors that influence predisposition to disease and markers that correlate with disease severity.^16–20^ An integrated analysis of insights obtained from multiple levels in the *omics* chain can collectively shed light on changes that are effectively translated from the genome and how they affect functions at the molecular level, thereby providing a comprehensive and integrated perspective on the myriad changes that occur upon infection, as well as on subsequent therapeutic intervention, paving the way towards personalised medicine.

Among the different levels in the host *omics chain,* exhaustive coverage has been provided by the increased availability of transcriptomic screens in TB across different populations as well as in multiple cell and tissue types, including patient whole-blood samples, isolated dendritic cells and neutrophils, and from macrophage cell lines exposed to *Mtb*.^21–26^ These studies have established that blood transcriptomics offers a robust approach for studying the immunology of TB and lends itself to integrated systems analysis to determine host responses in infection.^27–29^ The increasing availability of gene expression data across different geographical locations makes collective analysis of different datasets necessary in order to gain deeper insights into the host’s specific response to TB. However, such collective analysis is met with multiple challenges, and the measured signals in each study are heavily influenced by individual experimental design, depending largely on the sensitivity of the probes used in different platforms.^30, 31^ Heterogeneity among and between population samples studies coupled with experimental biases further compounds the problem of comparing different datasets describing TB.^32^ Transcriptomic studies of TB patients and their corresponding healthy controls shed light on differentially regulated genes [differentially expressed genes (DEGs)], and also permit monitoring of such differential regulation upon treatment. However, DEG-centric approaches cannot explain the underlying mechanisms behind the observed differential regulation by themselves, nor do they explain the functional consequences of such expression in a systematic manner.

Analysis of host networks provides a useful alternative to study molecular responses across multiple data platforms, enabling the integration of different types of *omics* data and providing a platform for visualisation and interpretation of changes triggered upon perturbations such as infection. The causes and consequences of differential regulation can be traced by monitoring the interactions of DEGs in the network. Previously, we established a methodology to construct response networks by integrating macrophage expression data into a protein–protein interaction network, and highlighted the ‘highest activities’ in the host macrophage upon infection with *Mtb*.^33^ While the pathway activities in the macrophage during infection provide insights into the initial innate responses of the host, the immune response to TB is complex and involves multiple players of both innate and adaptive immunity, including macrophages, dendritic cells, neutrophils, T cells, as well as several metabolic processes.^5, 15^ Whole blood captures a pool of immune cells that are trafficking to and from the sites of active disease and lymphoid organs, and serves as an easily accessible medium for analysis.

In this study, we describe a meta-analysis of multiple host response networks generated by integrating whole blood expression profiles from TB patients across different studies in order to find common disease-specific variations in an unbiased manner, resulting in the emergence of a unified network, which we refer to as the *common core,* reflective of the set of consistent changes in the host in TB. Such an approach overcomes platform-dependencies of individual microarray datasets, shifting focus from DEGs alone towards a more comprehensive analysis of the processes that are consistently altered, thereby shortlisting important molecular players that drive the host outcome upon infection. We observe the occurrence of the *common core* in an independent dataset, further strengthening the analysis. The core also shows variation in activity over the course of anti-tubercular therapy, and a comparison with the response networks of other inflammatory diseases such as pneumonia, sarcoidosis, Still’s disease, and systemic lupus erythematosus (SLE) reveals that the *common core* is a largely specific response to TB.

## Results

**Fig. 1:**
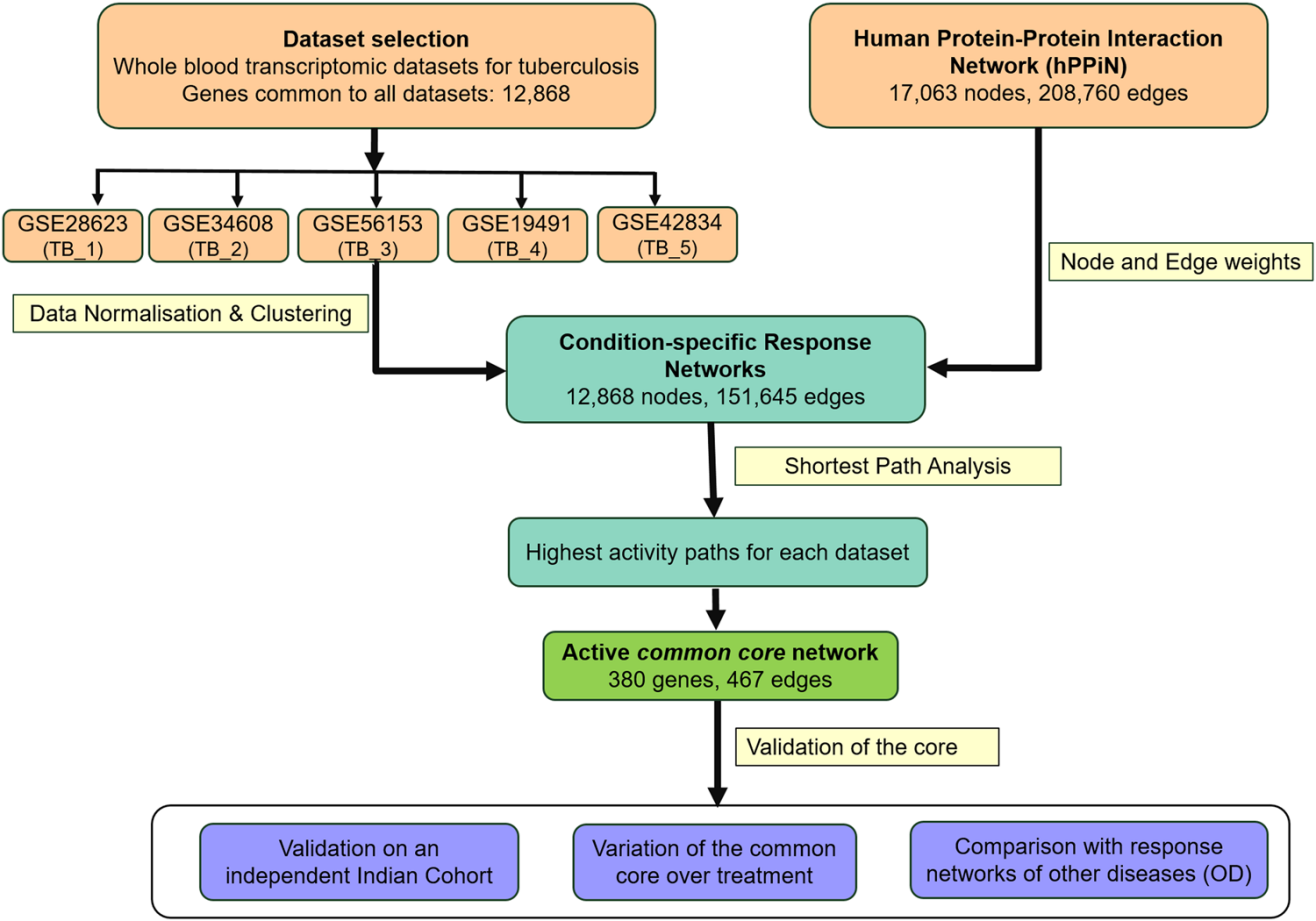
Workflow adopted in this study. Whole blood transcriptomic profiles from tuberculosis patients and corresponding healthy controls were normalised and integrated into a curated human Protein–Protein Interaction Network (hPPiN) to generate condition-specific networks, from which highest activity ‘response networks’ were identified. A comparison of these networks led to the identification of a *common core* highly active in disease. The significance of this *common core* was assessed on an independent microarray dataset generated for the Indian Cohort, and its specificity to tuberculosis was determined by monitoring its variation over treatment as well as by comparing similar response networks generated for other inflammatory diseases Sarcoidosis, Pneumonia, Still’s disease and SLE, collectively termed as OD

The overall approach implemented in this study and an overview of the results are illustrated in Fig. 1.

### DEGs show limited overlap across multiple datasets

**Fig. 2:**
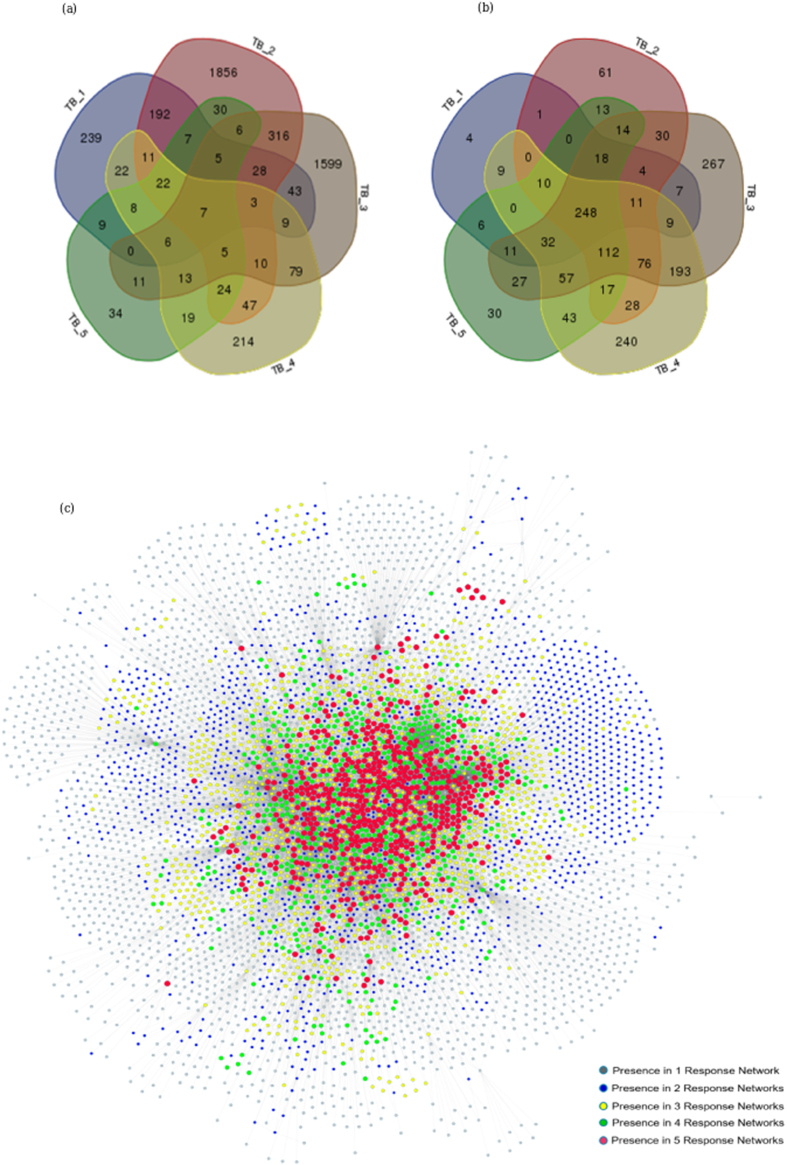
Comparison of whole blood transcriptomic datasets in tuberculosis at different levels reveals commonalities among differences. **a** A Venn diagram illustrating very little overlap between DEGs reported by individual datasets **b** Comparison of gene ontology terms for the DEGs however, reveal several common enriched biological processes across datasets **c** Pooled representation of individual response networks generated for all five datasets, illustrates a considerable amount of overlap. Node colours and sizes are proportional to the number of response networks the nodes occur in, with single occurrences seen at the periphery of the network (*grey*) and nodes occurring in all response networks (*red*) forming a highly interconnected core in the centre highlighting their high centrality

A total of five datasets describing host whole blood transcriptional expression in TB were used for meta-analysis, and are provided in Table 1. The transcriptomic datasets for meta-analysis were chosen such that they were from (a) whole-blood expression profiles generated through microarray experiments of (b) adult TB patients, (c) and have at least a few age-matched controls in the same dataset. Although there are a few more datasets listed for tuberculosis,^16^ several of them were from PBMCs and were therefore not considered to eliminate any bias due to differences in the source tissue. Those datasets that compared TB transcriptomes with other diseases, but not with healthy controls were also not considered. The 5 datasets chosen for this work have been utilised in several studies and are well cited, and have led to significant insights into the host response to tuberculosis, making them well suited for meta-analysis. The first question to ask of this data was to identify the set of genes that were consistently differentially regulated in TB patients across all datasets. Surprisingly, a comparison of DEGs across datasets showed very little overlap, with only seven genes commonly up-regulated or down-regulated across all five datasets considered (Fig. 2a). Such limited overlap could be a result of multiple factors, including differences in severity of infection, genetic heterogeneity among and between population groups analysed, as well as variations in the infecting strains in addition to platform-based dependencies for individual datasets.

**Table 1:** Transcriptome datasets used in this study. The number of samples selected reflect the samples chosen post normalisation and clustering in each condition

ID	Dataset	Platforms	Samples and condition studied	Population cohort	Reference
TB_1	GSE28623	Agilent	Whole blood from 46 TB and 37 HC	The Gambia	^23^
TB_2	GSE34608	Agilent	Whole blood samples: 8 TB, 18 HC, and 16 Sarcoidosis	Germany	^24^
TB_3	GSE56153	Illumina	Whole blood samples: 18 TB and 18 HC	London	^25^
TB_4	GSE19491	Illumina	Whole blood samples: 56 TB, 30 HC, 29 Still’s disease, 28 ASLE	UK, SA	^21^
TB_5	GSE42834	Agilent	Whole blood samples: 35 TB, 62 HC, and 13 pneumonia samples	London	^22^
TB_0, TB_2w, TB_2m, TB_4m, TB_6m	GSE40553	Illumina	Whole blood samples: 27 TB patients monitored at diagnosis, 2 weeks, 2 months, 4 months and 6 months post treatment.	SA	^40^

To understand the significance of such poor overlap despite a large number of DEGs reported in each study, we first carried out a clustering exercise for each dataset separately by considering the normalised signal intensity values for these DEGS in each patient and each healthy control individually within the dataset. A hierarchical clustering was then performed using standard protocols, which by and large yielded distinct clusters for healthy and TB samples in each case, replicating the observations reported in each study. Given this, we investigated if DEGs in different datasets represent the same set of biological processes and whether they represent different points of perturbation leading to the same functional destinations. We therefore performed individual gene set enrichment analyses for these DEGs, which revealed similar processes that are differentially regulated in TB across all datasets, implying that while each study may not report the same DEGs, they may highlight different DEGs belonging to common functional categories. (Fig. 2b). The DEGs computed for individual datasets, their overlap as well as the commonly enriched biological processes across these DEGs have been provided in Supplementary Table S1.

### Response networks capture disease-induced variations in an unbiased fashion

To test whether the processes enriched in disease across datasets are indeed related alterations, or if the different datasets have neighbourhoods of alterations that are common, we employed a networks-based approach and compared the top-ranked alterations in different datasets. First, a comprehensive curated, well annotated, global human protein–protein interaction network (hPPiN) was constructed, accounting for several signalling, metabolic and regulatory processes, thus providing a global coverage into the human protein interactome. The master network comprised 17,062 proteins (nodes) and 208,759 interactions (edges), of which 168,237 edges had an assigned direction based on their functional annotations, while the remaining 40,522 edges were considered bidirectional as they represent formation of structural complexes. The individual interactions in the network along with network statistics have been listed in Supplementary Table S2.

To obtain insights about the functional significance of variations in expression in disease, the base network was rendered condition-specific by mapping gene expression data in the form of node and edge weights in the hPPiN. Each node was assigned a weight proportional to the expression value of that gene in a particular condition, and the corresponding edge weights were inversely proportional to the weights of the node forming that edge, such that a lower-edge weight implied a higher expression value of the constituting nodes.^33^ Identification of high activities in these weighted networks was posed as a problem of computing shortest paths in each of the condition-specific networks. Shortest paths among all nodes in the networks were computed using Dijkstra’s algorithm, which computes a shortest path for each given source node to a target node by traversing the lowest-weighted edges in that route. Path cost is taken as the cumulative sum of the weights of the edges constituting the path (Eq. 4). Lower the path cost, higher is the perceived activity through that path. Since condition-specificity is brought about by variations in the weights on essentially the same blue-print, it is feasible to identify differences in routes or paths in the networks between healthy and disease. Differences in paths of high were thus mined in an unbiased fashion by identifying shortest paths with least path costs. The methodology utilising weighted networks has been previously demonstrated to be significantly robust to noise generated by minor variations in differential expression,^34^ and the rationale for computing weighted shortest paths has been discussed in Supplementary File S1.

Ten condition-specific networks were constructed, representative of five TB and five healthy conditions. In each of these networks, the set of paths representing highest levels of activities were first identified, which were then used to find active paths of highest difference in TB compared to their corresponding controls. We refer to these highest activity difference networks as ‘response networks’, since they reflect a systems view of the differential response observed in disease.

A total of 94,886,628 all-vs.-all shortest paths were computed for all conditions. The path cost formulation was devised such that paths with the least cost were considered to be most active, expected to contain highly expressed genes. A percentile-based ranking was adopted to rank paths, with lower cost paths attaining higher ranks. Two thresholds were considered to represent tiers of activity—paths in the 99.5th percentile were considered to be of highest activity (Tier-1), while those in the 99th percentile were considered to be of high activity (Tier-2), and were inspected for further analysis. While paths below this threshold could still be significant and their exclusion may result in the elimination of important genes, for purposes of identifying the most significant responses, we lay emphasis on the high activity paths alone, thereby erring on the side of caution. Both Tier-1 and Tier-2 paths were compared across the condition-specific networks for HC and TB, and paths unique to each TB network were considered. These unique highest activity paths in TB are now representative of the most active difference responses occurring in patients.

The nodes in the TB response networks expectedly comprise genes that are upregulated in disease, but in addition they also include those genes that are constitutively expressed and which form mediating ‘bridges’ among such upregulated genes, thereby providing mechanistic insights into the nature of the flow of such differential regulation in the host, which would otherwise be missed if the focus was on DEGs alone. Interestingly, we observe that the Tier-1 response networks for each TB dataset comprises a well-connected subnetwork of the hPPiN, implying that the differences observed are interrelated in some sense and possibly lead to a concerted set of variations as a collective response to disease.

Pooling the individual TB response networks by taking a union of the Tier-1 activities for individual datasets reveals the nature of the overlap among different datasets. The network topology is largely dependent on the degree or connectivity of each node, and nodes with a higher degree will be situated towards the centre of the network. As observed in Fig. 2c, the nodes present in only 1 dataset (*grey*) align at the periphery of the network, and increasing overlap of nodes in multiple response networks correlates with a more central positioning of the genes. The nodes that are present in all five response networks (*red*) are seen to be localised at the centre of the cumulative network, forming interconnected edges. The topological architecture of the pooled network thus points to a central core subnetwork of genes that is likely to drive the host responses in active tuberculosis, independent of the population cohort analysed. It is thus apparent that the DEGs in different datasets, although not identical, belong to common neighbourhoods in interaction networks. Analysis of such a subnetwork would thus lead to deeper insights into the primary processes that are regulated in the host in TB.

### Emergence of an active interconnected ‘*common core’* in disease

Comparison of different response networks reveals a higher overlap among nodes, in contrast to the comparison of DEGs alone, suggesting that despite limited commonalities observed at the individual gene level, the response networks can capture multiple similarities in biological processes and participating nodes. To identify the individual interactions driving such similar processes across datasets, the computed shortest paths for individual tier-1 TB response networks were compared, and a set of 713 common pathways was identified. These paths constitute an interaction network comprising 380 nodes and 467 edges, which we refer to as the *common core*. The *common core* is seen to be largely interconnected with only a few sparse edges, implying extensive cross-talk across multiple nodes that contribute to the molecular response in TB. To assess the probability of emergence of the *common core* out of chance, a permutation test was carried out as described in the Methods. The distribution of the overlap between randomly picked genes and the *common core* genes revealed that the maximum overlap with the *common core* in all the 10^^5^ random selections was 6%, clearly showing the probability of picking up these genes just by chance is very low.

**Fig. 3:**
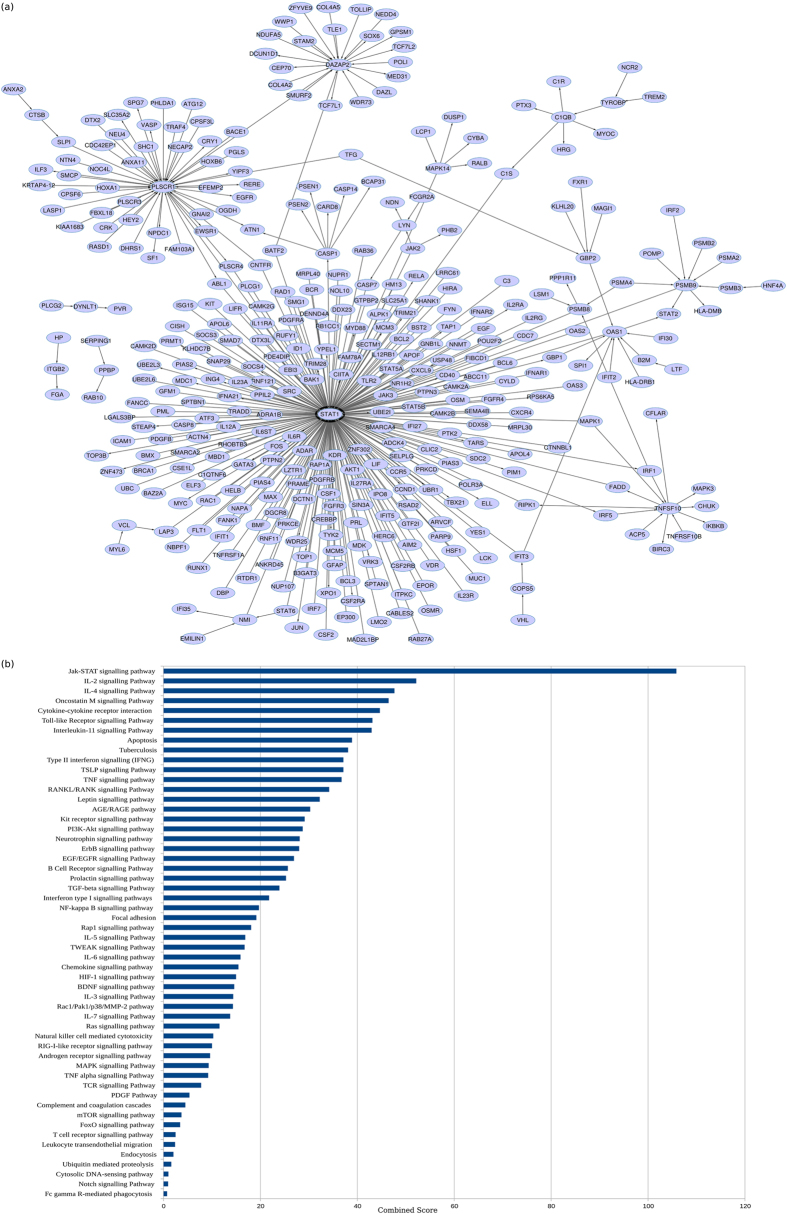
**a** The emergent *common core* characteristic of the host response in tuberculosis. **b** Pathway enrichment (*p* ≤ 0.05) highlights most significant biological pathways in the **a**

As seen in Fig. 3a, the *common core* is largely centred around *STAT1*, a transcription factor that primarily mediates pro-inflammatory responses as well as links other important signalling processes in the host. The other hubs in the network include genes *PLSCR1*, *DAZAP2*, *C1QB*, *OAS1* and *GBP2*. Response networks can thus capture common regulatory mechanisms across datasets encompassing multiple populations. The most enriched processes in the *common core* have been shortlisted in Fig. 3b. The *common core* interactions and detailed pathway enrichment have been enlisted in Supplementary File S3.

We observe the enrichment of several inflammatory processes involving signalling mediated by cytokines and their receptors, particularly by pro-inflammatory cytokines IL2, IL11, IL3, IL6, IL5, TNF and IFNG. Subsequently, the activation of anti-inflammatory processes mediated by cytokines IL-4 and TFG-beta is also observed. Complement and coagulation cascades are seen to be at play along with other signalling processes such as Kit receptor signalling and Notch Signalling pathways, as well as natural killer cells-mediated cytotoxicity, characteristic of tubercular infection. Cytoskeletal remodelling is actively observed, and can be attributed to structural changes in the cell during phagocytosis of *Mtb* along with leucocyte endothelial migration involved in the activation of T cell responses by chemokines secreted from macrophages and dendritic cells towards lymph nodes. While signalling processes are highly activated, host lipid signalling and metabolic processes are conspicuously absent in the *common core*. The subnetwork centred around *STAT1* involves multiple players that respond to both Type I and Type II interferons.^35^ *PLSCR1* is a calcium binding protein which is induced by interferons and several growth factors, and is known to mediate the movement of plasma membrane phospholipids in several processes including apoptosis and cellular injury.^36^ It is a significant contributor to the infection-triggered apoptotic responses observed in tuberculosis triggered by *CASP8*, and also serves as a receptor for the secretory leucocyte proteinase inhibitor *SLPI*,^37^ implicated in antimicrobial responses, which has been captured in the *common core*. *DAZAP2* is known to be influential in mediating Wnt signalling, and also participates in multiple signalling pathways^38^ including interactions with TGF-beta, a cytokine known to play a central role in curtailing inflammatory responses in TB. A* C1QB*-centred subnetwork highlights the activation of complement signalling. Several genes participating in the innate immune response are captured in the *common core*, including OAS1, *GBP1*, *GBP2*, *BCL6* and members of the TNF superfamily. While the *common core* consists of only 380 genes, these genes show a similar functional enrichment to that of the individual response networks containing significantly larger number of genes, implying that it is this *common core* that predominantly drives the relevant processes in the host in TB.

**Fig. 4:**
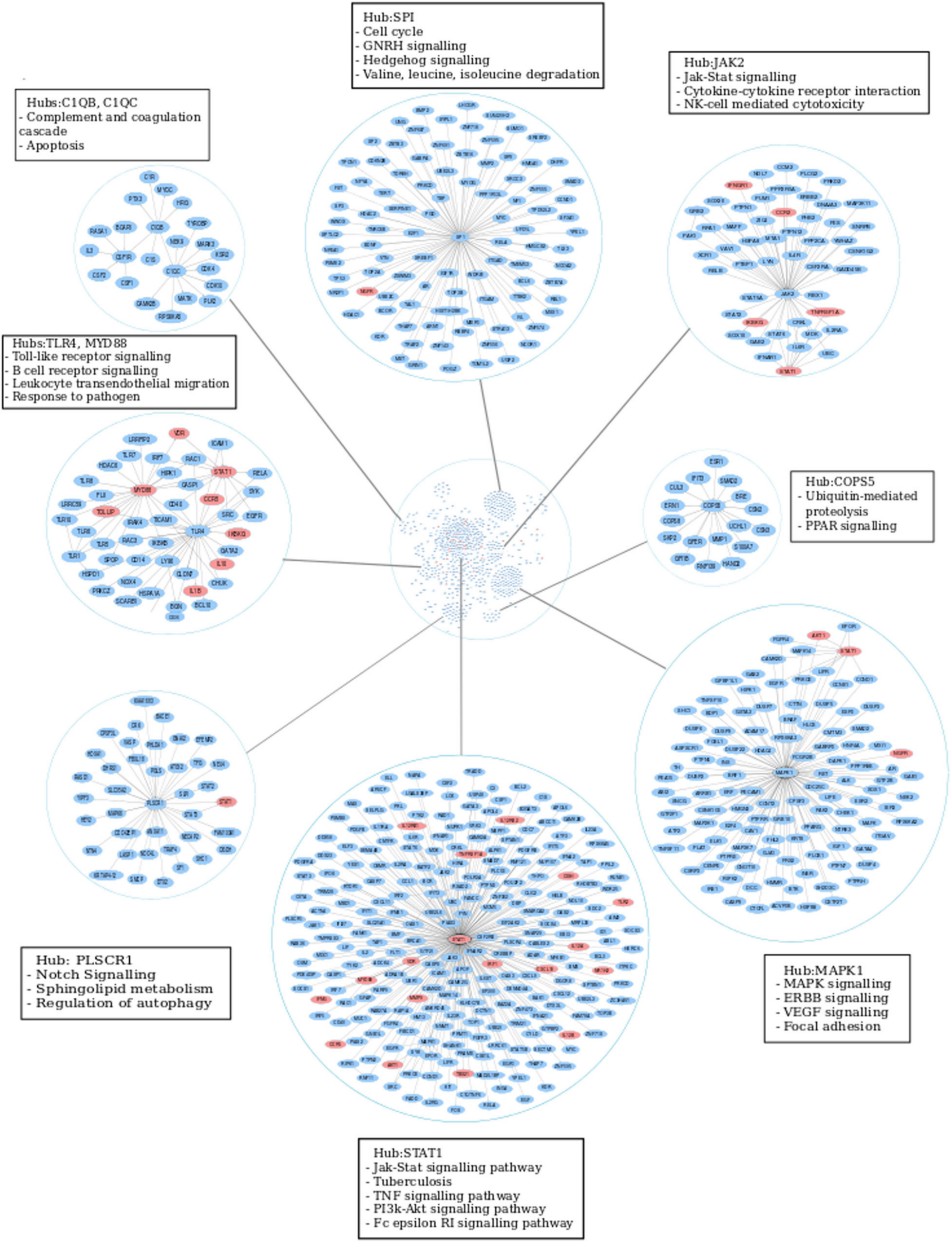
The Tier-2 *common core* depicting highly active nodes and their corresponding pathways enriched in disease. The *STAT-1* centric responses are retained at Tier 2 and the emergence of other well connected hubs such as *MAPK1 *and *SP1* is also observed, encompassing myriad signalling processes and their crosstalk across multiple cell and tissue types, captured in the whole blood milieu. Genes reported to have SNPs in different studies ascribing susceptibility to tuberculosis are marked in *red* in this network

To identify additional processes in the host, the tier-2 paths were compared across individual response networks, revealing an overlap of 1869 paths. These paths constituted a network of 747 genes and 920 edges (Fig. 4), largely retaining the topological architecture of the tier-1 *common core*. Additional subnetworks emerged around newer hub nodes *SP1*, *COPS5*, *JAK2* and *TLR4*, highlighting processes of immune activation and proteolysis. The topmost enriched KEGG pathways in each subnetwork around these hub nodes are also illustrated.

While transcriptomic changes provide insights into variations in gene expression, inspecting genetic polymorphisms reported by single nucleotide polymorphism (SNP) studies and genome-wide association studies in multiple populations in the context of response networks could depict how changes at the gene level are carried forward to result in variations in expression. Genes with genetic polymorphisms implicated with increased susceptibility to tuberculosis reported in literature^12^ and from the Online Mendelian Inheritance in Man database^39^ were enlisted, and 30 of these genes were seen to occur in the *common core.* These include *STAT1*, *CXCL10*, *TLR2*, *IRF1*, *RBBP8*, *VDR*, *IL10*, *IFNG*, *IFNGR1*, *AKT1*, *CCR2*, *CCR5, CISH, CYBB, IKBKG, IL12A, IL12B, IL12RB1, IL12RB2, IL1B, IRF8, MMP9, MYD88, NGFR, NR1H2, PAK2, TBK1, TBX21, TNFRSF1A,* and *TOLLIP*, and are highlighted in Fig. 4.

### The *common core* can sufficiently distinguish between diseased and healthy samples

**Fig. 5:**
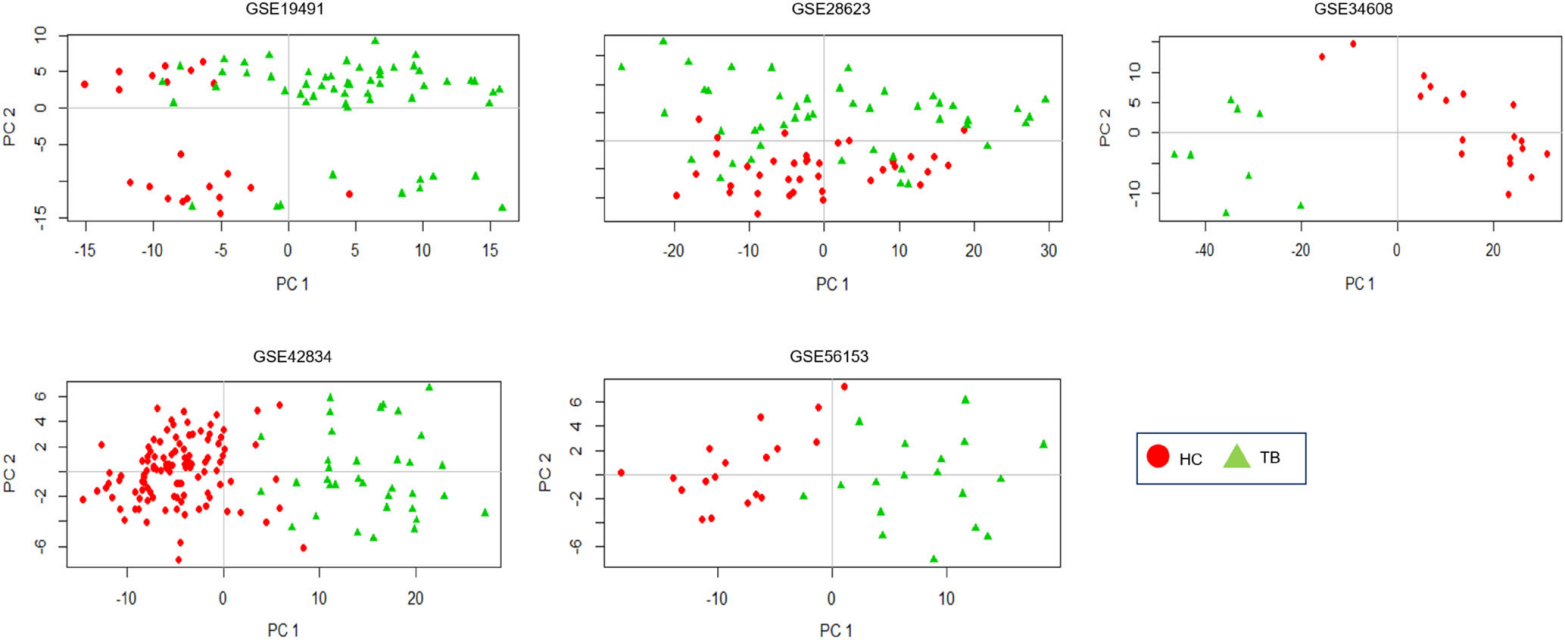
2-Dimensional Principal Component Analysis plots using the *common core* reveal significant separation between individual TB patients and HC samples across all datasets

To investigate if the *common core* was sufficiently characteristic of TB relative to healthy controls, we carried out a principle component analysis using the expression values of the genes constituting the *common core* from individual samples across all five datasets chosen for the meta-analysis. Figure 5 depicts the observed separation using the first two principal components of the *common core* across datasets, demonstrating the ability of the *common core* to yield a relatively a high extent of separation between healthy controls and diseased samples. This analysis serves to demonstrate that the *common core* can discriminate between the two conditions, laying further emphasis on its specificity to active disease.

### The *common core* exhibits partial reversal upon anti-tubercular treatment

**Fig. 6:**
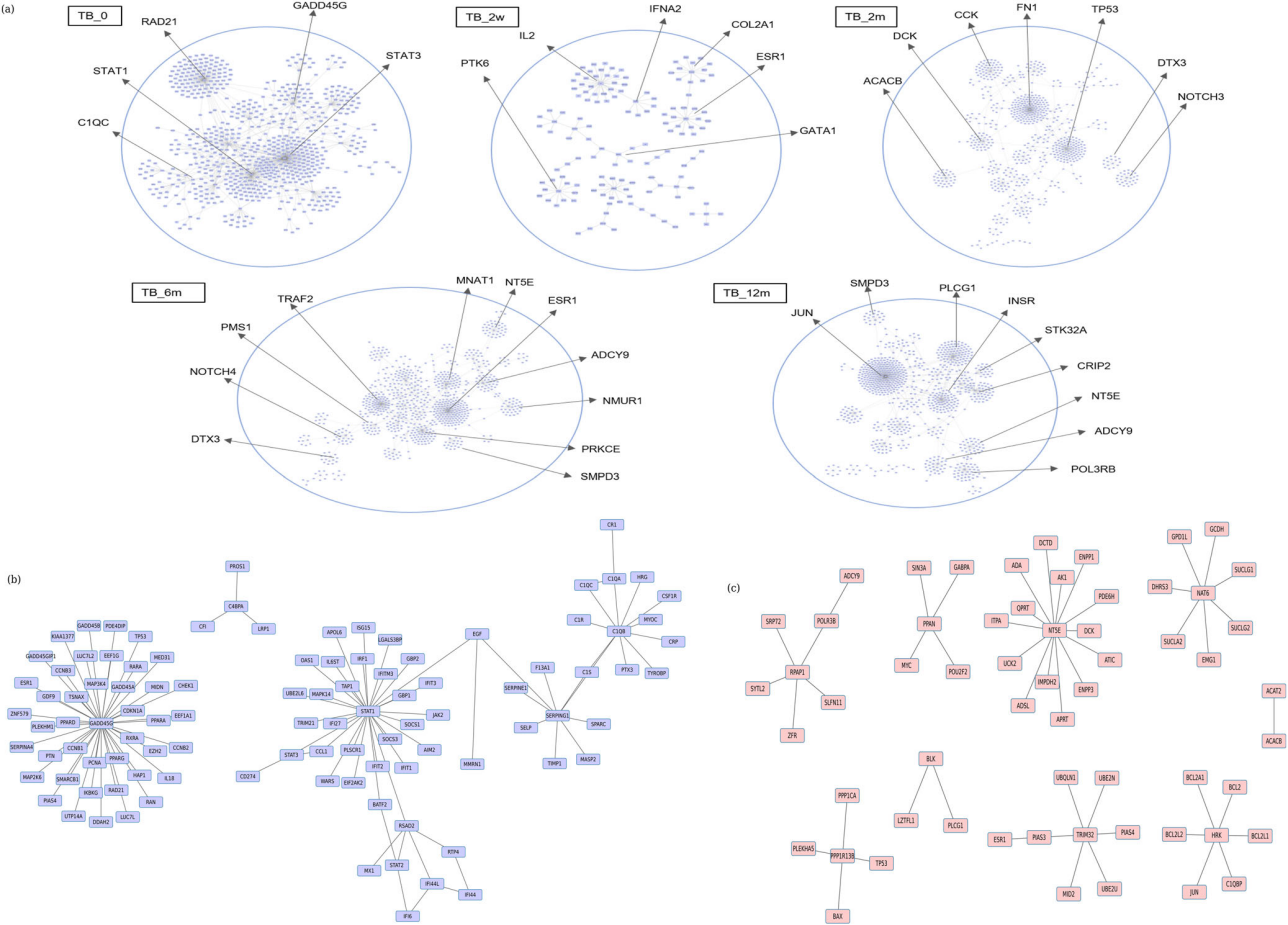
Monitoring the *common core* upon treatment. **a** Variation in network topology observed in individual response networks at time points of diagnosis, 2 weeks, 2 months, 6 months and 12 months post anti-tubercular therapy. The hub nodes occurring at different time points are highlighted **b** Subnetwork of the *common core* lost gradually over 6 months of treatment **c** Subnetwork emerging post 6 months of treatment indicating possible end points of therapy

As the above analysis is strongly suggestive of pathological relevance to the *common core*, we wanted to investigate if it showed variations over anti-tubercular treatment. Response networks were constructed from whole blood samples capturing expression profiles from patients subjected to standard anti-tubercular therapy.^40^ Samples were monitored at diagnosis of tuberculosis (TB_0), 2 weeks (TB_2w), 2 months (TB_2m), 6 months (TB_6m) and 12 months (TB_12m) post treatment. The response networks at different time points were constructed relative to TB_0. Analysis of these response networks revealed a change in the network topology within 2 weeks of treatment (Fig. 6a), with *STAT1* no longer being a central node, implying the reduction in inflammatory responses. Instead,* IL2*-mediated responses emerge as a significant hub, and the type 1 interferon responses are retained. The interactions mediating complement signalling are lost in TB_2w. The *common core* was observed at TB_0 but its presence witnesses a gradual disappearance over time, further strengthening the observation that the core is an infection-induced response in the host. Figure 6b reveals a subnetwork of the core that is completely lost after 12 months of treatment, and Fig. 6c reports the emergence of newer subnetworks around the hubs *NT5E, CRIP2, TRAF2, NOTCH4, MNAT, ADCY9, NMUR1, PLCG1, ESR1, PRKCB* and *PRKCG* upon completion of therapy, many of which emerge at time point TB_6m. In addition to these hubs, several interactions at TB_6m are largely retained at TB_12m, indicating that these genes could possibly reflect the end points of successful treatment. The individual response networks at different time points of therapy are provided in Supplementary table S4.

### A common repressed network highlights processes that are downregulated in disease

To determine the network of processes that are downregulated in TB, top-repressed networks were also constructed by considering all those paths that were downregulated in disease as compared to the controls, and a common repressed network was subsequently identified. This common repressed network is constituted by 125 genes, with 105 interactions between them. Contrary to the *common core*, this downregulated network is largely disconnected, with distinct modules forming around certain hub genes, as depicted in Supplementary Fig. S1a. Significant genes that mediate such downregulation include the chemokines *CCL7, CCL20, CCL25, RPS3, MYC, CYP2E1, CD19, ADCY8, FYN, IRF3, WNT1* and *WNT3*. The genes *PAK6* and *CRIP2*, which are present in this repressed network are also seen to emerge in the active response networks post anti-tubercular treatment, further strengthening the finding that these are indeed infection-induced downregulations which respond to treatment. Biological processes affected by these genes include Wnt signalling, Hedgehog signalling, signalling mediated by G-protein coupled receptors, cAMP signalling pathway, regulation of lipolysis, fatty acid omega oxidation, PPAR signalling pathway and tryptophan metabolism in the host, among others, as depicted in Fig. S1b.

### Validation of the *common core*

In order to validate the *common core*, we performed the following analyses—(a) analysis of transcriptome data from a fresh Indian cohort of TB patients and healthy controls to test whether the *common core* is consistent in these samples and (b) assessing the specificity of the core compared to other pathologically similar diseases.

### Comparison with a fresh cohort

To validate the significance and reproducibility of the *common core*, we sampled microarray data on an independent dataset from the Indian population. Whole blood samples were taken from five TB patients and two healthy controls, meeting the inclusion and exclusion criteria as described in the methods. Response networks were constructed and the subsequent high activity shortest paths and networks were analysed at Tier-1 and Tier2 to assess the extent of overlap of the *common core* in this dataset. The pathway enrichment for the tier-1 response network for this dataset revealed similar processes to the *common core,* including Type II interferon signalling, Oncostatin M pathway, cytokine and chemokine signalling including pathways mediated by *IL6, IL1, IL3, IL4, IL5, IL2*, TGF-beta and its receptors, antigen processing and presentation, phagocytosis mediated by Fc gamma receptors, and Fc epsilon RI signalling pathway, NOD-like receptor signalling, HIF1 pathway and other oxidative stress, among others. These DEGs in this response networks and the enriched pathways are provided in Supplementary Table S5. Analysis of the shortest paths at Tier-1 revealed an overlap of 453 paths with the Tier-1 paths constituting the *common core,* and a significant subnetwork of 291 genes out of the 380 genes in the *common core* was reproduced in this response network, and was seen to adopt a similar network topology as that of the *common core*, centred around *STAT1*. Other hubs that are also observed are *PLSCR1, DAZAP2, TNFSF10* and *C1QB*. Relaxing the threshold to tier-2 also showed an overlap of 1236 paths out of the 1869 tier-2 common paths generated by the meta-analysis. Such similarities and reproducibility of the core serve to further strengthen the significance of the approach. Supplementary Fig. S2 highlights the overlap between the genes in the *common core* and the corresponding tier-1 response network generated for this dataset.

### Specificity of the core

Several inflammatory diseases report a phenotype similar to tuberculosis with a marked inflammatory response, further impeding diagnosis of TB. To test the specificity of the *common core*, we compared it with the corresponding response networks of Sarcoidosis, Still’s disease, pneumonia, and SLE, which are collectively termed as other diseases (OD). The datasets selected for ODs are described in Table 1. Tier 1 comparisons of the OD response networks with the paths constituting the *common core* show little to no overlap, indicating that the *common core* is a largely specific TB response. There are 18 paths overlapping with TB and pneumonia, representing the *PLSCR1* hub and its interacting partners. Interestingly, while *STAT1* also emerges in the Tier-1 network in pneumonia, it makes a different set of interactions in pneumonia as compared to tuberculosis. Since it is the set of specific routes that constitute the top network for each condition, we focused on the similarities in these networks generated by common paths instead of the common nodes among the conditions. Further, additional similarities such as between the core and OD networks may emerge at lower thresholds, for purposes of analysis only the Tier-1 comparisons were considered, indicating specificity in the processes of highest activity in TB. The individual Tier-1 paths for ODs are presented in Supplementary Table S6, and the overlap between the *common core* and ODs are highlighted in Supplementary Fig. S3.

## Discussion

For several infectious diseases including TB there now exists extensive transcriptome data generated from individuals suffering from the same condition in geographically distant locations or in diverse cohorts and settings.^41^ Reconciliation of such data from diverse sources is becoming necessary to derive general patterns of disease biology for diagnosis, therapy or prevention in the future.^42^ A clear example of this difficulty is the observation of only 7 genes as common DEGs among the different cohorts of pulmonary tuberculosis patients. A DEG-centric approach alone may not necessarily be suggestive of disease pathology nor can it reveal general patterns of variation in the system. A network approach is useful to probe if the different sets of DEGs ultimately culminate in the modulation of the same functional modules. Monitoring the variations in the interacting partners of DEGs in the context of their interaction networks would facilitate such an analysis. Large scale networks typically used for studying biological systems can be broadly classified into two types—gene expression correlation networks and *protein–protein* interaction networks. The former, which is more frequently represented in current literature, reflect associations between gene-pairs whose expression patterns are correlated. The latter, on the other hand capture interactions between proteins that can lead to deciphering flows, of which biochemical or signalling pathways form classic examples. Approaches such as Weighted Gene Co-expression Network Analysis^43^ show the commonalities in modules between the different datasets but rely on networks based on co-expression patterns. The network reported in this study is based on high-confidence, physical interactions that have been experimentally verified bearing evidences in literature. A drawback of the protein-interaction networks, however, is that they are limited in coverage by the availability of interaction data. There could also be noise in the direction information, since directions may be condition-specific in some cases, whereas the networks will include data from multiple experiments. Nevertheless, the networks have been useful in obtaining a global perspective of variations in disease,^33^ leading to increased usage of protein interaction networks. Protein–protein interaction networks on their own suffer from the limitation of providing only a static picture of the system at a given time. However, integrating such networks with transcriptome or proteome data derived from patient samples for example, and if available over the course of disease or even treatment progression, will render the networks condition-specific, thereby providing a dynamic view of global changes in the system.

Networks intrinsically have the advantage of placing the nodes in their functional contexts and facilitating the tracing of pathways leading to a given biological goal. If different datasets contain variations in the participating nodes at different points in these pathways, they can be easily captured in the networks. In the *common core*, pro-inflammatory responses modulated by interferons via *STAT1*, and apoptotic responses among others, are highly enriched although the points of alteration may not be exactly the same across individual datasets. While the DEG overlap among different datasets was minimal, we observe the occurrence of several genes such as *AIM2* and *ID1* which are not upregulated in all datasets, and hence they do not intersect, but are present in the *common core* as they have a common regulator in *STAT1*. The approach can thus highlight common regulators that mediate infection-induced responses. It must be pointed out that the formulation does not impose connectivity at any stage.

Many studies have suggested the importance of Type-1 interferon system in response to TB. Similarly, the involvement of the *STAT1*-*VDR* interaction^44, 45^ and the importance of Complement Signalling have also been noted earlier. In addition, many new genes are identified in these networks that vary in two or more datasets and linked to the same functional modules. The networks are thus able to capture the entire set of perturbations in an unbiased manner in a single analysis. More importantly, the new finding in this study is the identification of these modules as the common minimal determinants of TB infection. Thus, a networks approach identifies a set of genes that are pathologically most important, providing important pointers for use in diagnosis and therapy. Upon anti-tubercular treatment, the *common core* shows variation, again emphasising their relevance to disease, and genes belonging to the common repressed network emerge in the response network 6 months’ post treatment, signifying reversals in activity. The *common core* is also fairly specific to TB and differs from the corresponding response networks generated for similar inflammatory diseases.

Many routes are identified by this approach, and some of these could be newly identified pathways, while many are rewired routes in disease as compared to healthy controls. Future studies which could follow up on some of these shortlisted routes in the laboratory and obtain some experimental support, would lead to the identification of newer biological pathways. Such an integrated approach thus provides a platform-independent foundation to attain maximum insights from available clinical data in a systematic, unbiased manner, and sheds light into the molecular characterisation of the host response to perturbations such as infection.

## Materials and methods

### Dataset selection and processing

Whole-blood microarray profiles of patients with active pulmonary tuberculosis with corresponding healthy controls were obtained from five different studies reported in the NCBI Gene Expression Omnibus (GEO) representing multi-platform data. Similar datasets were also available for TB patients monitored over the course of standard anti-tubercular treatment, and for patients diagnosed with sarcoidosis, pneumonia, Still’s disease and SLE. Background corrected files were processed by performing platform-specific normalisation in R using the package limma^46^ for Agilent data and lumi^47^ for Illumina datasets. All data was quantile normalised and log2 transformed. Post normalisation, individual datasets were subjected to hierarchical clustering using the package ‘hclust’ in R, utilising the Pearson’s correlation coefficient with default distance parameters (Euclidean) to detect sample outliers. The median values of control, disease and treatment samples were chosen to represent each condition in the dataset. Student’s t-test was employed to compute *p*-values for individual genes. Genes whose expression values changed by at least 2 fold as compared to the corresponding controls were considered differentially expressed. A *p*-value ≤ 0.05 was used to determine if the fold change for each gene was significant and consistent across all samples in each condition.

### Protein–protein interaction network construction

A comprehensive genome-scale human protein–protein interaction network was constructed and curated to include functional annotations and directions. High confidence, experimentally verified interactions were extracted from multiple protein–protein interaction sources and pathway databases, and additional interactions were mined from primary literature. Interactions of highest confidence having a combined score >900 were derived from The Search Tool for The Retrieval of Interacting Genes/Proteins (STRING) version 10.^48^ Regulatory interactions pertaining to transcriptional, post-transcriptional and pathway regulators were extracted from SignaLink 2.0.^49^ The Cancer Cell Map^50^ contains information about genetic and physical interactions among genes and their products in any cell, and how these interactions are impacted by alterations in the genes. This map was queried to identify interactions present in non-diseased conditions in the host. Further, the BioGRID database^51^ was mined to identify additional unique interactions not reported by the other resources used. In addition to PPI databases and resources, primary literature was explored to identify experimentally verified interacting proteins in the human proteome. Interactions were extracted from a study by Khurana and co-workers^52^ who reported the construction of an integrated human PPI network termed ‘Multinet’, and from the macrophage interaction network constructed by Sambarey *et al.* ^33^ The functional nature of the interactions was identified by surveying literature as well as from network repositories such as GeneMania^53^ and the ‘protein actions’ file from STRING to assign directions to the edges accordingly. Functional annotations of interactions such as ‘activation’, ‘inhibition’, ‘phosphorylation’, ‘proteolysis’ and ‘ubiquitinylation’ were considered to determine the nature and direction of each edge. Some interactions described a physical binding event, and were considered as bidirectional. Additionally, those interactions which had high confidence scores and experimental evidence but did not have any functional annotation were also kept as bidirectional. All interactions were combined to give a unified, curated and high confidence human protein–protein interaction network which we report as hPPiN.

### Weighing the network to form condition-specific response networks

The normalised intensities for each condition and corresponding healthy controls were mapped on to the curated protein–protein interaction network in the form of node and edge weights, to generate condition-specific response networks. The formulations are adapted from the study published by Sambarey *et al.* ^33^ The node weight for node *i* in a diseased condition A is computed as:

1 $${N}_{i(A)}=F{C}_{i(\frac{A}{B})}\times S{I}_{i(A)},$$

where FC is the fold change of gene *i*, SI the normalised signal intensity of the gene, *A* the diseased state, and *B* the healthy control. For the TB response networks, FC for gene *i* in TB was computed with respect to HC, while the FC for HC response network was kept as 1. The antilog values of the respective signal intensities were used to compute fold changes.

The edge weight in a given condition for edge *e* in condition *A* (*W*_e(A)_) comprising nodes N_*i*(A)_ and *N*_*j*(A)_ is computed as:

2 $${W}_{{e}_{ij}(A)}=Inverse\sqrt{{N}_{i(A)}\times {N}_{j(A)}}\,$$

To generate repressed networks, the node weight for node *i* in condition *A* with respect to its corresponding control (condition *B*) was computed as:

3 $${N}_{i(A)}=F{C}_{i(\frac{B}{A})}\times S{I}_{i(B)}\,$$

### Shortest path analysis

Shortest paths were computed for the individual response networks by considering all nodes, using the Dijkstra’s algorithm, implemented in python. The algorithm computes minimum weight shortest paths, in which each path begins from a source node and ends with a sink node, through interacting proteins, choosing the least-cost edge in every step. The rationale For a path of length *n* in condition *A*, the path cost was computed as a summation of the edge weights constituting the path. The resultant shortest paths were ranked-based on their path cost using a percentile approach, with paths having a lower path cost given a higher rank.

4 $${\rm Path\ cost} = {\sum _{e=1}^{n}} {W_{e(A)}}$$

### Network visualisation

All networks were visualised in Cytoscape version 3.3.^54^ Network properties were computed using the NetworkAnalyzer plugin for Cytoscape, and the Allegro Spring-Electric layout was selected for representation of the network topology.

### Gene and pathway enrichment

Gene set enrichment analysis was implemented using the Panther database to identify significantly enriched Gene ontology biological processes. The EnrichR server^55^ was used to identify significantly enriched KEGG Pathways and WikiPathways, which were pooled and ranked based on the combined score.

### Permutation test

A permutation test was performed to assess the extent of overlap between response networks expected by chance, as described in ref. 56. The same number of genes present in the *common core* were randomly picked 10^^5^ times to obtain 10^^5^ different random gene-sets. The extent of overlap between these random sets and the *common core* were compared, with the null hypothesis that there is no difference between random gene-sets and the *common core*. The *p*-value for this comparison was computed as:

$$p=\,\frac{No.\,of\,{R_{\rm g}} >0.1}{No.\,of\,permutations}$$

where *R*_g_ is a random node-set from the generated graphs.

### Dataset generation and sample processing

For purposes of validation, independent whole-blood samples were collected from freshly diagnosed TB patients in the Indian Cohort, from the SDS-TRIC Rajiv Gandhi Institute for Chest Diseases, Bangalore. The patients had to be 18 years and above, first-episode, sputum tested positive for tuberculosis with the diagnosis confirmed by a pulmonologist. The exclusion criteria included absence of any other form of tuberculosis or co-morbidities such as HIV, chronic bronchitis, diabetes mellitus, cancer, sarcoidosis or Cytomegalo Virus infection. Pregnant women, lactating mothers and those on any co-medication were also excluded. Written consent was obtained from all patients.

### RNA extraction and microarray analysis

Total RNA was isolated from whole blood samples using the Ribopure RNA isolation kit (Ambion Inc.) according to the manufacturer’s instructions. RNA was quantified using a NanoDrop-1000 spectrophotometer. The microarray experiment was carried out using Agilent platform 039494 from Genotypic Technology. The scanned images were analysed with Feature Extraction Software 10.7.3.1 (Agilent) using default parameters to obtain background subtracted and spatially detrended Processed Signal intensities. The data was processed using the limma package in R where it was log2 transformed and quantile normalised.

### Availability of data and materials

The computational pipeline adopted in this study to generate response networks is available on GitHub (https://github.com/AbhinandanD/RNG.git). Gene expression data from the Indian Cohort has been deposited on NCBI GEO and is available with the identifier GSE81746.

## Electronic supplementary material

Supplementary Information

Supplementary Figure 1

Supplementary Figure 2

Supplementary Figure 3

Supplementary Table 1

Supplementary Table 2

Supplementary Table 3

Supplementary Table 4

Supplementary Table 5

Supplementary Table 6
