# Supplementary material for: Meta-analysis of host response networks identifies a common core in tuberculosis
Source: NPJ Syst Biol Appl. 2017 Feb 10;3:4. doi: 10.1038/s41540-017-0005-4 (PMC5445610; doi:10.1038/s41540-017-0005-4)
Supplement: Supplementary file 1 — Supplementary Information [file 41540_2017_5_MOESM1_ESM.docx]

**Rationale for using weighted networks** - The base human protein-protein interaction network captures only the network topology and will remain the same in any given condition of health or disease. This is not useful for capturing condition-specific information. To render it condition specific, we weight the network (by weighting the nodes and edges) using condition-specific data. Whole blood transcriptomes are the most comprehensively covered genomic data that are reflective of such condition specificity. Many studies have shown that transcriptomes when clustered by themselves, segregate into distinct clusters for healthy samples and disease sample respectively. However, they are inadequate by themselves to indicate which genes are important and why. Condition-specific networks overcome that problem efficiently and are hence used here, using a methodology that has been previously established in the laboratory [1]. Other network analysis techniques have been used which includes identification of hubs, densely connected components and control points. These techniques are useful for unweighted networks as they capture features related to network topology, and are thus not emphasized on in this manuscript since they do not depict variations between different conditions, such as between healthy and diseased samples.

**References**

1. Sambarey A, Prashanthi K, Chandra N: **Mining large-scale response networks reveals 'topmost activities' in Mycobacterium tuberculosis infection**. *Sci Rep* 2013, **3**:2302.
