# Supplementary figures and images for: Meta-analysis of host response networks identifies a common core in tuberculosis
Source: NPJ Syst Biol Appl. 2017 Feb 10;3:4. doi: 10.1038/s41540-017-0005-4 (PMC5445610; doi:10.1038/s41540-017-0005-4)

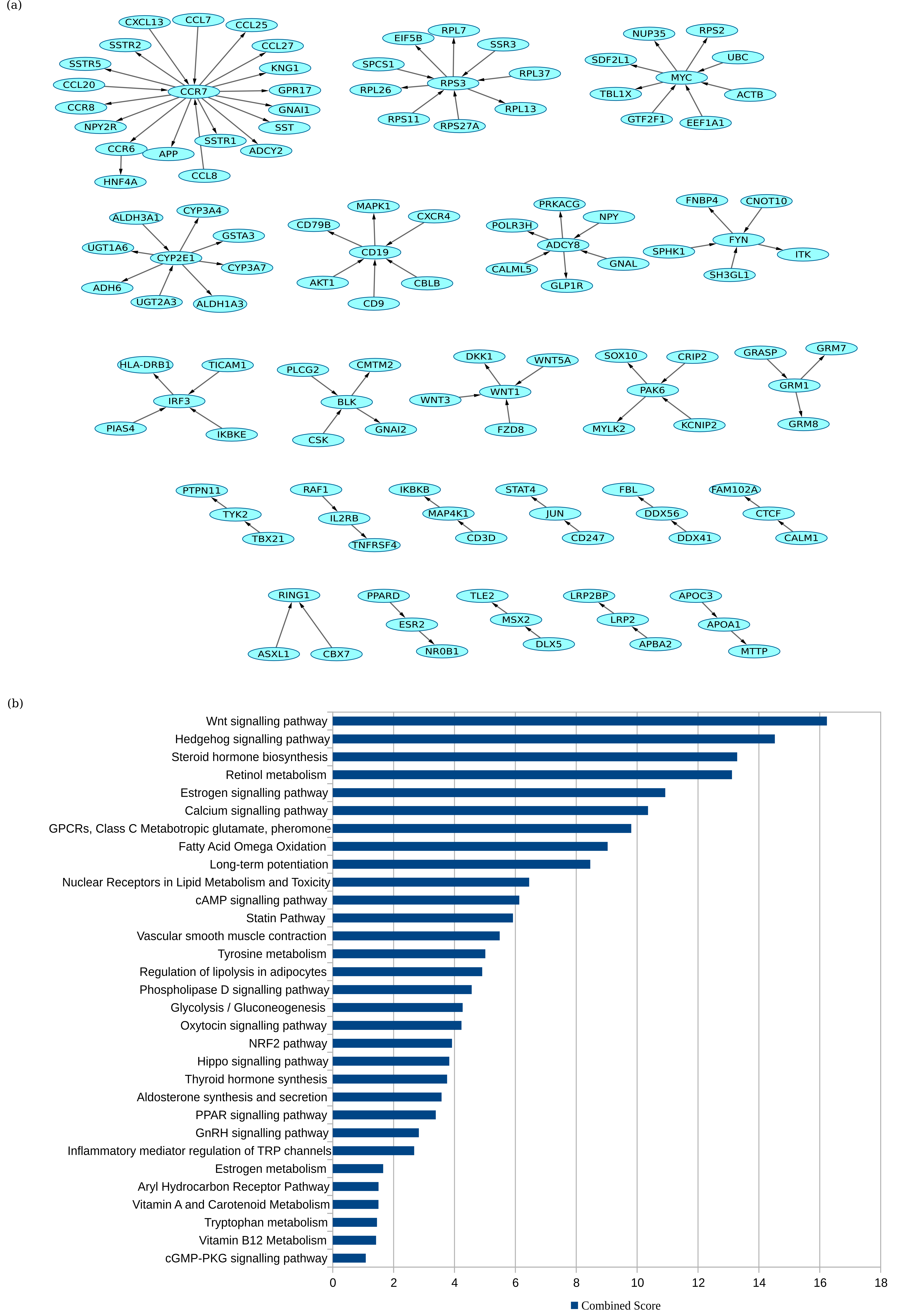

Supplement: Supplementary file 2 — Supplementary Figure 1 [file 41540_2017_5_MOESM2_ESM.tif]

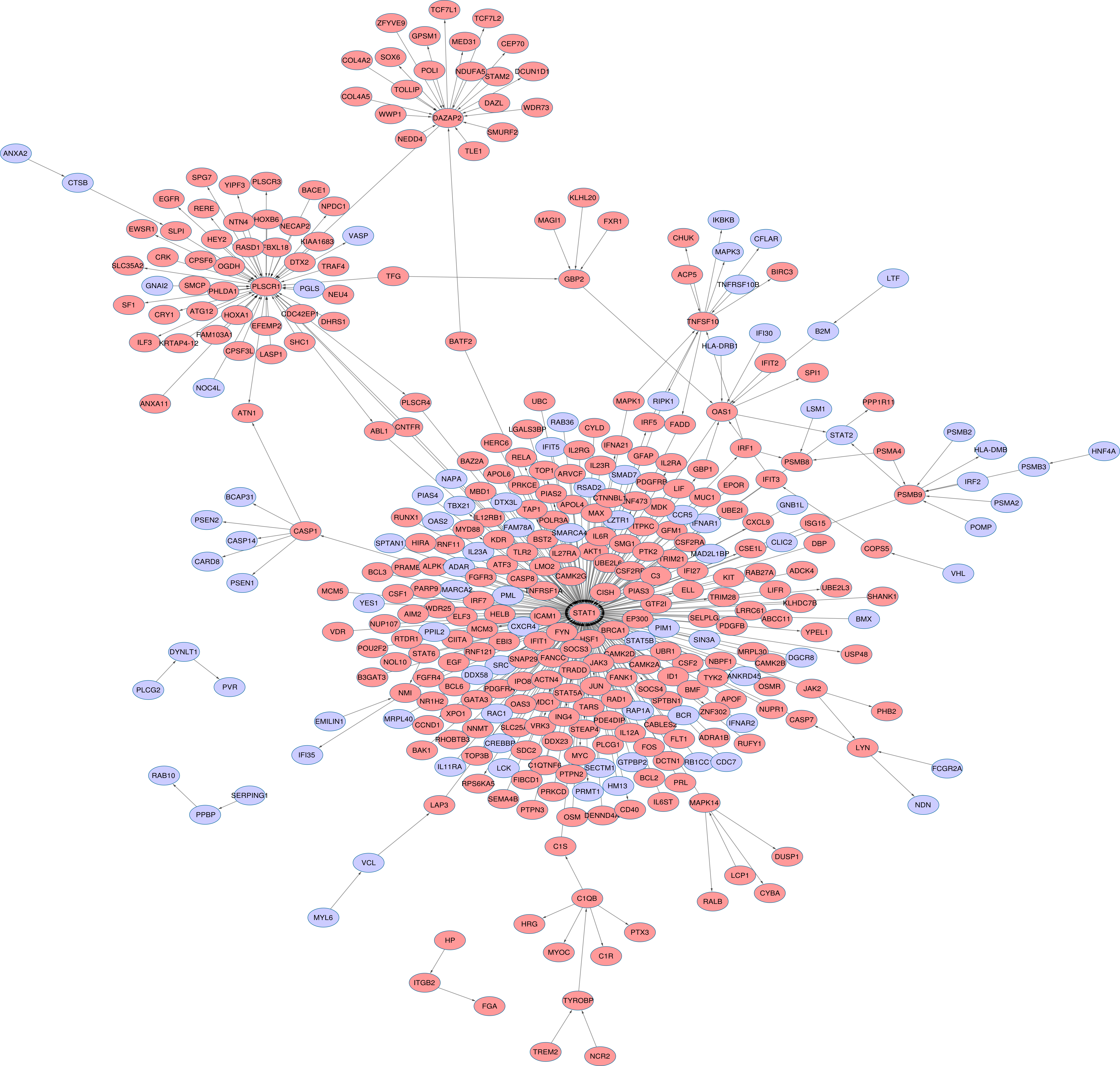

Supplement: Supplementary file 3 — Supplementary Figure 2 [file 41540_2017_5_MOESM3_ESM.tif]

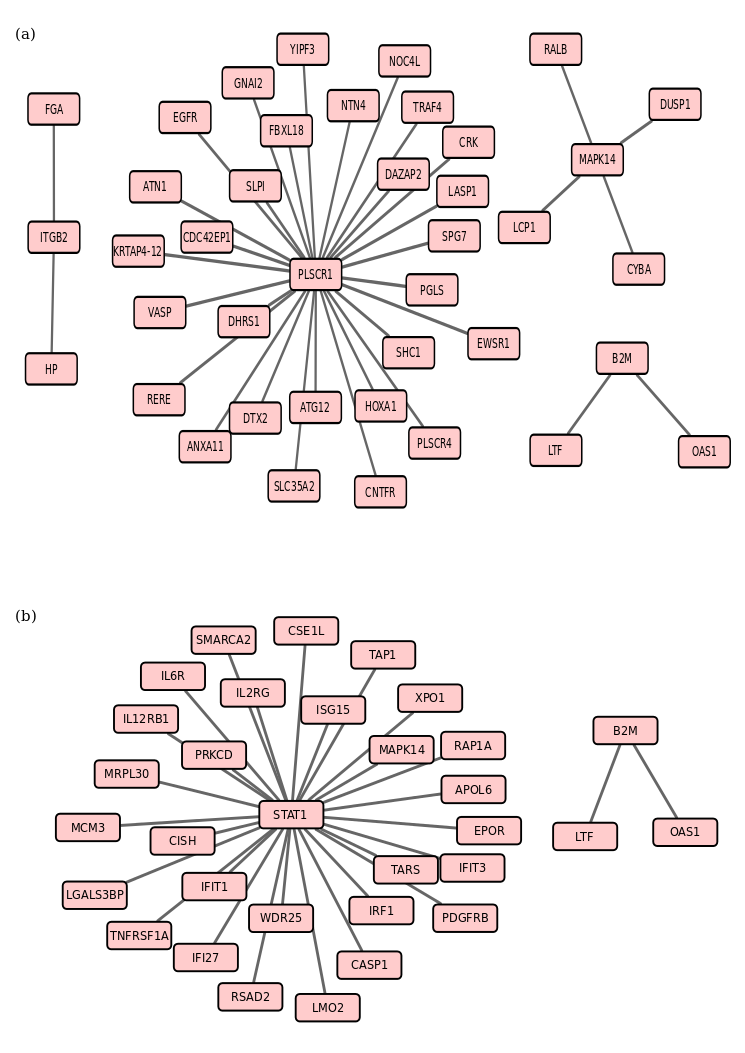

Supplement: Supplementary file 4 — Supplementary Figure 3 [file 41540_2017_5_MOESM4_ESM.tif]
